# Comment on “Sedative and Analgesic Effects of Entonox Gas Compared with Midazolam and Fentanyl in Synchronized Cardioversion”

**DOI:** 10.1155/2016/3834891

**Published:** 2016-03-15

**Authors:** Henrique Horta Veloso

**Affiliations:** Evandro Chagas National Institute of Infectology, Oswaldo Cruz Foundation (FIOCRUZ), Rua Conde de Bonfim 255, Sala 505, Tijuca, 20520-051 Rio de Janeiro, RJ, Brazil

In the recent investigation of Masoumi et al. [1], the intravenous association of midazolam and fentanyl was compared to inhaled nitrous oxide in the synchronized cardioversion. This study is very important since several drug schemes have been used for anesthesia during electrical cardioversion, with different mechanism of action, duration, and side effects, but, until now, no technique demonstrated a clear superiority over one another [2].

The primary endpoint of Masoumi et al. [1] was the degree of pain experienced by the patient, and secondary endpoints were sedation duration, time to full recovery consciousness, and need of additional doses to induce and maintain sedation. After randomization and analysis of 40 patients, this investigation demonstrated that nitrous oxide promoted a better analgesic effect and that it was also associated with shorter sedation duration and time to full recovery consciousness. In spite of these important observations, this study presented some limitations.

Initially, patients were included if they presented with “tachydysrhythmia associated with symptoms requiring cardioversion (unstable tachydysrhythmia with palpable pulse regarding patient conditions).” This criterion may include both ventricular and supraventricular tachyarrhythmias. Unfortunately, the authors did not present neither the diagnosis of the tachyarrhythmias nor the underlying heart disease of the patients treated. These data are extremely important to establish the safety of the drug schemes and its future choice according to the clinical profile of the patient. In a previous series [3] with inclusion criteria similar to the commented on study, the proportion of patients with ventricular tachycardia was of 13%. The majority of the studies with anesthesia for electrical cardioversion have included cases with atrial fibrillation in an elective procedure [2, 4], a more stable and predictable scenario.

Furthermore, the successful conversion rates with synchronized direct current shock should be presented too. It was clear that this was not an endpoint; however, this information is too much important to be missed. In some rare cases, anesthesia may restore normal sinus rhythm before shock discharge [5–7], and also, it is not definitely established whether the technique of anesthesia influences the defibrillation threshold [8–10] and consequently the efficacy of the cardioversion procedure or, at least, the number of shocks and energy required.

In conclusion, I do believe that studies investigating anesthesia drugs for electrical cardioversion must present the diagnosis of the tachyarrhythmia treated, the underlying structural heart disease of the patients, and the success rates of the procedure.
